# Nutritional Risk Factors Model of Community-Dwelling Older People in Poland–Pilot Study

**DOI:** 10.3390/nu17132150

**Published:** 2025-06-27

**Authors:** Robert Gajda, Marzena Jeżewska-Zychowicz, Karolina Rak, Monika Maćków

**Affiliations:** 1 Department of Human Nutrition, Faculty of Biotechnology and Food Sciences, Wrocław University of Environmental and Life Sciences, Chełmońskiego 37, 51-630 Wroclaw, Poland; karolina.rak@upwr.edu.pl (K.R.); monika.mackow@upwr.edu.pl (M.M.); 2 Department of Food and Consumption Market Research, Faculty of Human Nutrition, Warsaw University of Life Sciences, Nowoursynowska 166, 02-787 Warsaw, Poland; marzena_jezewska_zychowicz@sggw.edu.pl

**Keywords:** nutritional risk factors, questionnaire, sociodemographic determinants, economics determinants, older people

## Abstract

Nutritional risk factors are country-specific and change over time, requiring systematic verification. Objective: The study was designed to develop a nutritional risk factors model for seniors living in a Polish community. Methods: The pilot study was conducted in 2022 and 2023 among 301 people aged 60 and older in the Lower Silesia region of Poland. The questionnaire contained 107 test items describing dietary problems rated on a five-point Likert scale. The pre-study concerned understanding of the test items, rating the reproducibility (kappa statistic) and reliability of the scale (α-Cronbach coefficient). The factor structure of the model was developed using structural equation modelling (SEM) in the program R (version 4.3.2.). An exploratory factor analysis (EFA) extracted the three-factor model. Results: The factors were described as unhealthy eating (eight test items), irregularities related to meals (four test items), and perception of body weight (four test items). The model was verified using confirmatory factor analysis (CFA). The model’s acceptability was confirmed based on data matching indexes, convergent accuracy, differential accuracy, and measurement reliability. There was variation in the identified nutritional risk factors by gender, education, social activity, and family relationships. Conclusions: Focusing on irregularities in nutrition and perception of body weight as nutritional risk factors reveals a very narrow perspective in diagnosing nutritional risk, thus further testing of the model, in a representative group of older people in Poland and other countries, is necessary to confirm the results obtained.

## 1. Introduction

Globally, the number and proportion of adults aged 60 and older are increasing [1]. By 2050, the number of people aged 65 and over will be around 1.5 billion, meaning that one in six people worldwide will be of this age [2]. In Poland, the subpopulation of older people aged 60 and over in 2023 accounted for 26.3% of the total population, and the proportion has been growing steadily yearly since 2006 [3]. As the population ages, the prevalence of chronic diseases and multimorbidity will also increase [4,5]. For older people, non-communicable diseases are the main health risk [6]. Until recently, cardiovascular disease, cancer, and type 2 diabetes accounted for 11 million diet-related deaths [7]. In addition, 255 million attributable disability-adjusted life years (DALYs) could be traced back to nutritional risk factors [8].

Human eating behaviour changes over time as it is linked to the life cycle, and the environment in which it operates [9,10,11,12,13]. The reasons relate to changing demographic, sociocultural, psychological, globalisation, climatic, and epidemiological conditions over time [9,10,11,12,13]. Improvements towards healthy eating behaviours could prevent one out of five deaths worldwide [7]. However, changes in these behaviours can contribute to identifying various nutritional problems, which experts define as nutritional risk factors [14,15]. Among the global population, the most commonly identified dietary risk factors are low intake of vegetables, fruits, whole grains, and dairy products [7,16,17]. The consumption of these food groups is below the theoretical minimum risk exposure level (TMREL), which minimizes the risk of all causes of death [7]. Diets lacking in vegetables (<290–430 g per day), fruit (<200–300 g per day), whole grains (<100–150 g per day) and dairy products (<350–520 g per day) cause 65% of all diet-related deaths and 72% of all diet-related DALYs [18]. The need to identify new risk factors was confirmed during the COVID-19 pandemic [11]. At that time, excessive food consumption and consumption of highly processed food were identified as risk factors [19,20,21,22]. Among the older people, the problem is even greater. The increase in risk factors in this age group is related to the changes in the body and state of health [23] and to the lower socioeconomic status of older people [24]. Among the most commonly reported nutritional risk factors noted in the literature are as follows: change in body weight due to improper diet [25,26,27,28]; food intake that differs from dietary recommendations for older people [25,29,30,31]; problems with over- or under-consumption of food supplements and meal replacements [25,28,30,32]; problems associated with poor nutrition resulting from various functional disorders of the gastrointestinal tract [25,26,27,33]; nutritional problems linked to lack of social support [25,26,34,35]; problems with access to food [25,26,34]; problems with food preparation [25,26]; and change in appetite due to medication [25,26,27].

It is essential to eliminate nutritional risk factors in the lives of older people. Studies have shown that healthier eating habits are associated with better individual health outcomes, such as reduced risks of all-cause mortality, frailty, cardiovascular diseases, chronic conditions, type 2 diabetes, and neurodegenerative diseases [36,37,38,39,40,41,42]. Healthier eating habits, such as increased consumption of fruits and vegetables, can also lower the risks of depression, anxiety, central obesity, and cognitive health issues like Alzheimer’s disease in older adults [43,44,45,46,47]. Conversely, older adults with lower dietary diversity are more likely to experience anxiety or loneliness [48,49]. Despite the consensus on the definition of malnutrition, there is still no distinction between nutritional risk and malnutrition risk [50]. While the risk of malnutrition refers to individuals with indicators of malnutrition, such as very low food intake, weight loss, and abnormal functional and health parameters, nutritional risk refers to conditions and factors related only to a reduced quantity, increased quantity, or inappropriate quality of food consumed. Over time, failure to address these conditions or factors can lead to malnutrition or overweight-related health problems [25].

Older people living in the community may have problems with food consumption and therefore may be vulnerable to nutritional risks [28,30,51,52,53]. This can lead to poorer health and higher mortality rates [27,28,29,30]. According to a worldwide study using the SCREEN-14 questionnaire (Seniors in the Community: Risk Evaluation for Eating and Nutrition), the problem of high nutritional risk among older people (age 65 and older) affects 61.5 to 70.1% [54], and individual and environmental factors determine these risks.

The nutritional risk is more widespread than the risk of malnutrition and is most likely to affect community-dwelling older people in Poland as well [55]. Based on the SCREEN-14 questionnaire, a recent study found that as many as 77.5% of Polish older people living in the community are affected by high nutritional risk [56,57]. In this context, the study focused on what nutritional risk factors occur in older people living in local communities in Poland and how sociodemographic and economic factors differentiate the occurrence of nutritional risk factors. This study aimed to develop a nutritional risk factors model of community-dwelling older people in Poland, considering selected sociodemographic and economic characteristics.

## 2. Materials and Methods

### 2.1. Study Design and Sample

The pilot study was conducted between December 2022 and December 2023, with participants aged 60 and older. A non-probability sample selection was used. The choice of this method was based on the fact that it is cost-effective and helps to recruit reluctant individuals to the study. The references from known individuals helped recruit the study group. Through the Wrocław Center for Social Development, clubs, foundations, and other senior citizen organizations in the city of Wroclaw and in several counties of the Lower Silesian province—Wrocław, Świdnik, Trzebnica, and Sieradz districts—were asked to participate in the study. A total of 301 questionnaires were distributed to twelve senior citizen organizations, including six in the city of Wrocław (n = 183), one each in the districts of Trzebnica (n = 12) and Sieradz (n = 10), and two in the districts of Świdnica (n = 64) and Wrocław (n = 32). The study was conducted in small groups in the presence of one of the authors, who thoroughly explained and supported respondents in completing the questionnaires. The questionnaire was completed in person by the respondents. In case of any difficulties, the study’s author completed the questionnaire. The recruitment criteria were age 60 and over and residence in the community. The exclusion criterion for the study was the lack of informed consent to participate in the study. The recruitment principles for the study is presented in Table 1. Ultimately, 301 respondents aged between 60 and 94 (71.6 ± 5.77) participated in the study, including 241 women and 60 men.

The study was conducted under the guidelines presented in the Declaration of Helsinki [58]. Participation in the survey was voluntary. Informed consent to participate in the study, permission to publish the study results, and permission to process personal data for scientific purposes were obtained from all participants. The Bioethics Committee of the Medical University of Wrocław approved the study on 21 December 2022, opinion number: KB-912/2022.

### 2.2. Questionnaire

The questionnaire was developed to identify nutritional risk factors based on the available literature on nutritional risk among older people. The questionnaire contained 107 test items describing nutritional risk factors [25,26,27,28,29,31,34,35,59]. The test included the following items: changes in body weight over the past 6 months and perceptions of body weight (items 1–6); nutritional problems, i.e., food intake and nutrition from the dietary recommendations recommended for the older people according to the guidelines of the National Center for Nutrition Education in Poland [31] (items 7–60); consumption of dietary supplements and meal replacements (items 61–75); problems associated with food consumption due to various functional disorders of the gastrointestinal tract (items 76–84); problems with access to food (items 85–89); problems with food preparation (items 90–92); problems with food consumption for social reasons (items 93–103); and changes in appetite related to drug intake (items: 105–107). A 5-point Likert scale was used to assess nutritional risk situations, with a range of responses: no (1), rather not (2), neither no nor yes (3), rather yes (4), yes (5).

Questions on gender, age, place of residence, education, social activity, family relationships, family and social financial support, personal financial situation, and household economic situation were used to characterize the study group and to differentiate nutritional risk factors in sociodemographic and economic terms, which were taken from the KomPAN questionnaire [60].

To prepare the questionnaire for the study, a pre-study consisting of two stages was conducted:

Pre-testing was conducted to assess understanding of each test item. Sixteen people at a selected senior citizens’ club in Wrocław were asked to participate in the pre-testing phase. A 3-point scale was used to assess understanding of the description of the situation, with the following answers: I understand (1 point), I partially understand (2 points), I do not understand (3 points). This was followed by a panel discussion among the same people, which focused on their understanding of the situation and suggestions regarding the misunderstood content. For each item, a mean score of 1.5 or more (X ≥ 1.5) indicated misunderstanding. One hundred seven items were tested, among which eighty-eight were incorrectly understood. Based on this information and the results of the discussion panel, 10 test items were removed from the questionnaire. Other items were corrected grammatically, logically, and linguistically. The revised questionnaire (97 test items) was retested with a group of 17 people in a selected senior citizens’ club in the Świdnica district using the same rating scale and the criterion for understanding the description of the item (X ≥ 1.5). This time, all items were understandable, considering the adopted criteria.

In the next stage, the reproducibility and reliability of the questionnaire were assessed. At a selected senior citizen club in Wrocław, 93 people were asked to fill out a questionnaire and, after 3 weeks, to fill it out again (retest). To verify the measurement accuracy of the tested variables, a reliability analysis was performed using Cronbach’s alpha method [29]. The Cronbach’s alpha reliability coefficient for the scale (97 test items) was estimated at α = 0.88 (M = 2.10; SD = 0.41) and showed that this measure’s reliability had a high measurement accuracy. Cohen’s kappa statistic was used to assess the questionnaire’s reproducibility. The questionnaire achieved excellent agreement by 7 test items, good by 37, moderate by 27, and poor by 26. All sixteen items included in the model of nutritional risk factors described in the Results section had at least moderate agreement, which was considered acceptable.

A description of the nutritional risk items included in the study questionnaire (97 test items) is provided in the Appendix A (Table A1).

### 2.3. Statistical Analysis

Descriptive statistics were used to show the sociodemographic and economic characteristics of the sample.

The reliability of the questionnaire for identifying nutritional risk factors of Polish seniors was tested using Cronbach’s alpha coefficient, with a value higher than 0.70 considered acceptable [61,62]. The concordance of the test items (test–retest) of the questionnaire was assessed using the kappa statistic (Cohen’s kappa index). Concordance of test items is very good when the kappa statistic is ≥0.81, good (0.61–0.80), moderate (0.41–0.60), poor (0.21–0.40), and bad (≤0.20) [63,64,65]. A kappa index of at least 0.41 was considered acceptable.

To develop the factor model of the nutritional risk, structural equation modelling (SEM) was carried out in the program R and using the package “lavaan” [66]. The ULSMV (unweighted least squares mean and variance adjusted test statistic) algorithm was used for the calculation. ULSMV is often the preferred estimation algorithm due to its ability to handle ordinal data (e.g., Likert scale) that does not meet the assumptions of normal distribution [67].

Exploratory factor analysis (EFA) with varimax rotation with Kaiser normalization allowed confirmation of the factor structure. The following criteria were used to determine the number of factors: an eigenvalue of 1.0, a scree plot test, and factor loadings of at least 0.45. The Kaiser–Meyer–Olkin (KMO) measure of 0.780 and Bartlett’s sphericity test at *p* < 0.0001 confirmed the factoriality of the data [68].

The fit of the factor structure identified during the EFA was tested using confirmatory factor analysis (CFA). The following model fit indices were evaluated: chi-square/degree of freedom (χ^2^/*df*), comparative fit index (CFI), Tucker–Lewis index (TLI), Bollen’s incremental fit index (IFI), root mean square error of approximation (RMSEA), standardized root mean square residual (SRMR), goodness-of-fit statistic (GFI), and adjusted goodness-of-fit statistic (AGFI). Acceptable values of the listed parameters are χ^2^/*df* below 2 or 3; CFI ≥ 0.95; TLI ≥ 0.95; IFI ≥ 0.95; RMSEA < 0.06; SRMR < 0.01; GFI and AGFI, the closer the value is to 1, the better the explanation of the theoretical model by the data [62,69,70,71].

To test the model using SEM analysis, the direction of influence (→) of the emergent factor (latent variable) on the test items was determined, and the determination coefficients were calculated (*R*^2^) [72].

The test items trafficities in the model were assessed using two indicators: AVE (average variance extracted) to assess convergent accuracy and HTMT (heterotrait–monotrait ratio) to assess differentia accuracy. AVE > 0.50 indicates that the assumption of convergent accuracy is satisfied. HTMT ≤ 0.80 suggests that the assumption of differential accuracy between measurements is met. The reliability of the measurement was assessed using Cronbach’s alpha index and CR (composite reliability). Cronbach’s alpha coefficients and CR > 0.70 indicate acceptable measurement accuracy [73,74,75].

Two categories were identified within each factor: low factor intensity and high factor intensity. The median for the factorial values was used as the cut-off point. For factor 1 (F1), Me = −0.023, factor 2 (F2), Me = −0.140, and factor 3 (F3), Me = −0.120, with low intensity < Me and high intensity ≥Me. The chi^2^ test determined the differences between factor intensity and selected sociodemographic and economic characteristics. A *p*-value < 0.05 was considered significant.

## 3. Results

### 3.1. Characteristics of Study Sample

The sociodemographic and economic characteristics of the study group are shown in Table 2. The study sample consisted of 301 respondents, represented mainly by women (80.1%), people aged 60–74 (72.8%), and residing in Wrocław city (60.8%), the district of Świdnica (21.2%), or the district of Wrocław (10.6%). The most significant number of respondents had secondary education (50.5%), average financial status (80.1%), and declared no need for family (81.4%) or social financial support (86.0%).

### 3.2. Structure of the Factorial Model

The structure of the factor model is shown in Table 3. The EFA analysis resulted in three factors described as the following: factor 1 “unhealthy eating”, factor 2 “irregularities related to meals”, factor 3 “perception of body weight”. Factor 1 (F1) concerns dietary situations related to low dietary variety, limiting or omitting healthy foods, and eating unhealthy foods simultaneously. This factor identified a low frequency of vegetable consumption (less than twice a day) and a high frequency (daily) of consuming highly processed foods (powdered soups, canned or jarred, refrigerated, and frozen foods). This factor also included opinions on the difficulty of choosing healthy foods when shopping. Factor 2 (F2) was related to situations involving incorrect number or frequency of meals, too few meals (less than three meals per day/breaks between meals longer than four hours), or too many meals (more than six meals per day/breaks between meals shorter than two hours). Factor 3 (F3) considered situations related to weight change and included subjective assessments indicating too low weight (I think my weight is too low/would like to increase my weight) or too high weight (I think my weight is too high/would like to decrease my weight).

### 3.3. Characteristics of Factorial Model

**Figure 1:**
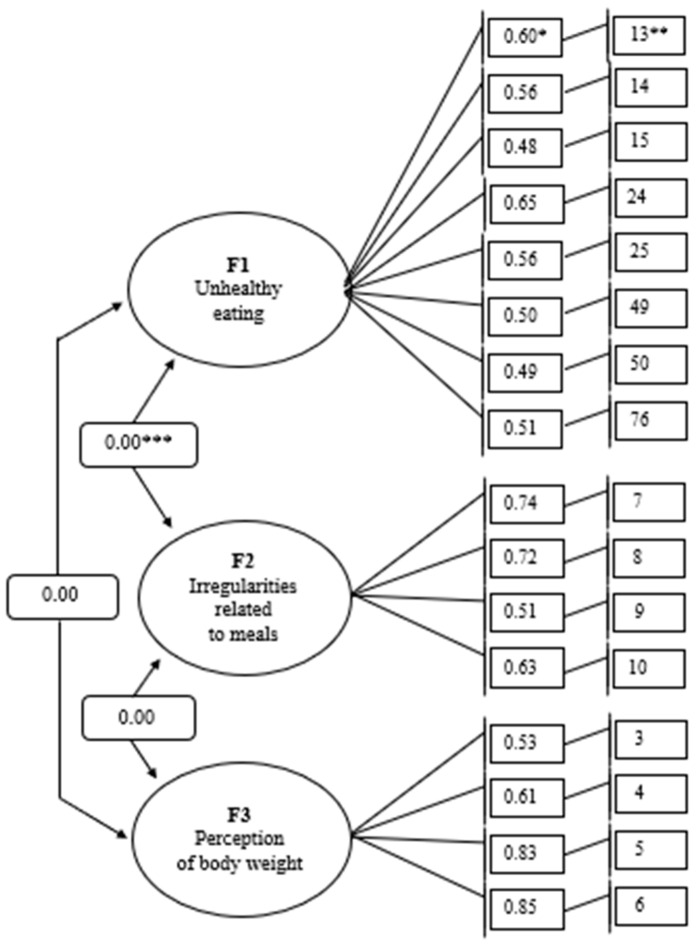
Model of tested variables with correlation of factors. * standardized factor loading (β); ** test item number; *** the value of the correlation coefficient (R).

The EFA and CFA resulted in a factor model with the correlation of factors shown in Figure 1. All test items included in the selected factors obtained acceptable values of standardized factor loadings (β > 0.4). The CFA was also used to estimate model fit indexes (Table 4). The values of the CFI, TLI, NFI, IFI, RMSAE, and SRMR measures indicate a relatively good fit of the data to the tested model. The values of the GFI and AGFI measures ranged from 0.93 to 0.95. The closer the values are to 1.00 for the GFI and AGFI measures, the better the explanation of the theoretical model by the data. The chi-square test result did not meet the conditions for fitting the data to the tested model, slightly exceeding the acceptable criterion (χ^2^/*df* = 3.34).

SEM analysis showed that all the emerging factors significantly impacted the variables tested in the model. In addition, the SEM analysis showed that none of the emerging factors had their non-specific indicators. The direction of influence of the emergent factors in the model on the variables tested and an assessment of the predictive power of the factor model are shown in Table 5. Test items such as: “I think my body weight is too low” (*R*^2^ = 0.73), “I think my body weight is too high” (*R*^2^ = 0.69), “I eat three or fewer meals per day” (*R*^2^ = 0.55), “I eat six or more meals per day” (*R*^2^ = 0. 52), “I eat raw vegetables and fruits less than twice a day” (*R*^2^ = 0.43), “The gaps between my meals are longer than four hours” (*R*^2^ = 0.40), are explained by the model by at least 40%. The model explained the remaining test items by at least 24%.

Verification of the assumption of measurement reliability showed that Cronbach’s alpha (internal reliability) and CR coefficient (construct reliability) met this assumption for all factors in the tested model. Verification of the assumption of meeting convergent accuracy (AVE) showed that only factor 3 met the assumptions (Table 6). On the other hand, verifying the assumption of differential accuracy (HTMT) showed that factor 1 and factor 2 measure different information, similarly, factor 1 and factor 3, and factor 2 and factor 3. Thus, HTMT analysis meets the assumption of differential accuracy for the models tested (Table 7).

### 3.4. Sociodemographic and Economic Determinants of the Nutritional Risk Factors

Age, place of residence, family and social financial support, personal financial situation, and household economic situation did not differentiate the intensity of nutritional risk factors. High intensity of factor 1 (F1) related to unhealthy eating was more common in men. As education increased, the proportion of people with a high intensity of this factor decreased, but it improved as family relationships deteriorated (Table 7). A greater frequency of various social activities and poorer family relationships were associated with high factor 2 (F2) intensity involving meal irregularities. High intensity of factor 3 (F3) related to perceived body weight was more common among women, those with higher education, higher social activity, and poorer family relationships (Table 8).

## 4. Discussion

The identification of nutritional risk factors currently experienced by community-dwelling older people in Poland was driven by two main reasons: 1/changing nutritional risk factors over time, which requires monitoring [9,10,11,12,13]; 2/the higher exposure of older people than other population groups to nutritional risks and associated health consequences [27,28,29,30,51,52,53]. This study aimed to identify nutritional risk factors in community-dwelling older people, considering selected sociodemographic and economic characteristics.

To achieve the aim, the model of nutritional risk factors was developed, and then the validity and reliability of the model were assessed using structural equation modelling (SEM). The model fits the data because values of fitness indexes, i.e., indexes of absolute fit (RMSEA and GFI) and incremental fit (CFI and TLI) achieved acceptance. Only the index parsimonious fit (χ^2^/*df*) slightly exceeds the fit criterion [76,77]. By the recommendation, using at least one fitness index from each model category confirms the model’s fitness [78], allowing the model to be accepted. The model’s validity was assessed using convergent, construct, and discriminant validity. The convergent validity was achieved because all items in a measurement model were statistically significant [79]. However, a construct “perception of body weight” obtained the value of AVE higher than 0.50, which was expected [74]. The construct validity was achieved because the values of absolute fit indexes (i.e., RMSEA and GFI) and incremental fit indexes (i.e., CFI and TLI) confirmed it. The discriminant validity was also achieved because the measurement model was free from redundant items. Moreover, the HTMT ratio was less than 1.0, indicating that the constructs have good discriminant validity [75]. The internal consistency of the model, estimated by the Cronbach’s alpha index, is considered adequate for each one of the factors because its value was greater than 0.6 [80]. The construct reliability was confirmed by a value of CR ≥ 0.6, which is required [74]. Although the indicators obtained are satisfactory, it should be noted that the model was developed on data from only a specific sample. Thus, the findings in this study cannot be generalized because the testing of the model is limited to the sample examined in this study. Therefore, further research is needed to confirm the usefulness of this model [81].

In the identified model, one of the factors is related to nutritionally adverse situations, including those associated with poor dietary variety, limiting or omitting healthy foods from meals while consuming unhealthy foods, low frequency of vegetable consumption, and frequent consumption of highly processed foods. Previous studies, including Polish seniors, have reported the poor quality of the diet in a significant proportion of the elderly population [57,82,83], unfavourable dietary patterns [1,58], and/or low diversity of food intake [84,85]. Eating behaviour, dietary patterns, and diet quality are determined by several factors [1,84,86]. Among the differentiating factors are gender [86,87,88,89,90,91,92]; age [1,87,89,91,92]; education [84,86,88,89,91,92]; economic situation [1,86]; and social situation, including family and social support, loneliness, social isolation, and family relationships [93,94,95,96,97]. In addition, socioeconomic status, which usually refers to residence, education, social support, and economic situation, plays a crucial role [1,24,82,84]. In this study, age, place of residence, family, social and financial support, personal financial situation, and household economic situation did not differentiate the unhealthy eating factor. The association between age and eating behaviour patterns in older people is inconclusive [1]. While age was negatively associated with a “vegetable-based” pattern [87], no relationship was observed between age and the “Mediterranean” or “healthy” pattern [91,92]. In another study, a “healthy” nutritional pattern was negatively associated with age group 75 years or older in a group of women. At the same time, no association was observed in the younger age group [89]. Similarly, in the case of economic and social factors, the situation seems unclear [1,86,93,94,95,96,97]. Some studies show no relationship between income and “healthy” nutritional patterns in older people, others show a positive relationship [1,86]. In the case of social factors, while several studies have assessed the relationship between various social characteristics and dietary patterns, they have observed a different relationship [93,94,95,96,97]. Higher levels of socioeconomic status tended to be associated with more favourable diets of older people [24,82,84]. Zhu et al. [98] showed that people of low socioeconomic status tend to lead unhealthy lifestyles, including eating foods of low nutritional value. The results confirmed that the intake of foods beneficial to diet quality (vegetables, fruit, whole grains, milk, and fish) increased with higher socioeconomic status [56,98].

Research indicates that older women have a more favourable diet than men [86,87,88,89,90,91,92]. In this study, the high severity of the factor associated with unhealthy eating was more common among men, which may have been justified by the findings reporting that older women are more likely to be associated with a “healthy” or “Mediterranean” nutritional pattern [89,91,92]. It was associated with higher consumption of vegetables and pulses, fruit, cereals, potatoes, fish, seafood, and dairy products [86]. Furthermore, while habitual vegetable intake among older people was too low and is associated with risky eating behaviours, women consumed more vegetables. They were less likely to engage in risky eating behaviours than men [1].

In published studies, lower education was associated with “unfavourable” nutritional patterns or risky eating behaviours [1]. In this study, higher education was more often associated with a low intensity of factor 1 and vice versa for lower education. This finding can be confirmed by other studies, in which higher education among older people was associated with a more favourable diet, mainly a higher intake of vegetables, fruit, whole grains, and dairy products [86,88,89,91,92] and greater diversification of food consumption [84].

A similar relationship was noted for family relationships. Very good relationships were associated with low severity, and poor relationships were associated with high severity of the unhealthy eating factor. Living with a family or in a care institution was associated with more favourable eating patterns than living alone [97]. Older people characterized by close family or friendship relationships were shown to be less able to guide their preferences when choosing food [99], which may have resulted from the excessive caring function of family or friends for older people. In contrast, older people with weakened family ties were shown to have a greater capacity to be guided by their preferences. Still, the consequence of this capacity was food choice, determining lower diet quality [99].

Unhealthy eating was associated with the frequent consumption of highly processed foods by older people, mainly concentrated and semi-prepared foods or ready-to-heat meals, which can be explained in several ways. The study was conducted during the period following the lifting of the COVID-19 pandemic measures in Poland [100], which may have influenced changes in dietary behaviour [101] related to the consumption of highly processed foods [19,20,21,22]. Moreover, in the case of older people, access to this type of food was made easier, if only because of the offer of small chain and neighbourhood shops located near their residence [102,103,104], with high interest during the pandemic.

A greater frequency of various social activities and poorer family relationships were associated with high irregularities related to meals. The higher frequency of meal intake was mainly among older people eating out, especially men and those of higher socioeconomic status [105]. On the one hand, the higher social activity of older people may determine a higher frequency of eating out and therefore consuming more meals. On the other hand, high social activity may limit the time spent eating out and thus reduce the number of meals. Older people’s consumption of meals at home increases the regularity of their meals. It optimizes the number of meals consumed, with women and those with better socio-economic status being more likely to do so [105]. Poorer diet quality is related to the number of meals, lower meal quality, poorer perception of meals, and poorer enjoyment, which is seen primarily among older people living alone [106,107]. These negative dietary experiences are often seen among single men than women [106]. In contrast, eating meals by older people in the family or in the company of others was associated with higher energy and nutrient intake due to higher consumption of cereal products, meat, oils and other fats, vegetables, fruit, and mushrooms [108]. While we do not see studies evaluating the relationship between family relationships and the number and frequency of meals consumed, indirect information [106,107,108] may suggest that good family relationships among older people may influence better meal structure related to the number and frequency of meals consumed, but this requires further research.

Factor 3 was related to weight perception and included subjective assessments indicating that body weight was too low or high and a declared desire for its increase or decrease. The dynamic nature of the ideal body image, which is influenced by the changing prevalence of overweight and obesity over time [109], implies corresponding fluctuations in self-perceived body weight [110]. Consequently, research to date indicates an increasing tendency for people to identify themselves as overweight or obese [111,112,113]. A recent study found that older people perceive their body weight to be too high regardless of their actual body weight or when this was not the case [114,115]. Older people’s perceptions of their body weight consistently reveal a tendency to underestimate their actual weight, in contrast to the younger subpopulation [116,117,118,119,120]. Despite this, there is a noticeable gap in existing research on weight perception among older people [115]. Research indicates that older people’s perceptions of their body weight being too low are overlooked. Only one study found that older people, regardless of gender, who perceived their body weight as too high or too low had higher rates of poor health [121]. In this study, the weight perception factor was more common among women, those with higher education, higher social activity, and poorer family relationships. Previous research indicates differences regarding older people’s perceptions of body weight, but only in the context of gender. It was shown that it was predominantly women who undertook weight reduction efforts who perceived themselves to be overweight or obese, regardless of actual body weight [114,115].

### 4.1. Limitations of the Study

Although the study was carried out with a high degree of rigour, pre-verifying the survey instrument using a discussion panel, relevance and reliability tests, and using the best statistical tool for factor model design, the SEM analysis [67,122], this study has several limitations. Above all, the study is cross-sectional—the causal relationship between risk factors and their determinants and the changes in these factors over time cannot be assessed. In addition, the non-probabilistic sampling does not make it possible to relate the results to the entire population of older people in Poland. The risk factors involve subjective measures that rely on participant self-reporting, which may limit the accuracy of results [123]. Due to the peculiarities of the study group in which the model was tested and the limitations indicated earlier, it is suggested that the study be conducted again to confirm its relevance [124].

### 4.2. Practical Application of the Study

The procedure used in the study allowed for the inclusion of a large number of items describing various situations of older people that may constitute nutritional risk factors. These include change in body weight due to improper diet [25,26,27,28]; food intake that differs from dietary recommendations for older people [25,29,30,31]; problems with over- or under-consumption of food supplements and meal replacements [25,28,30,32]; problems associated with poor nutrition resulting from various functional disorders of the gastrointestinal tract [25,26,27,33]; nutritional problems linked to lack of social support [25,26,34,35]; problems with access to food [25,26,34]; problems with food preparation [25,26]; and change in appetite due to medication [25,26,27]. The use of modelling allowed us to reduce the number of risk factors, which can help monitor the problem of nutritional risk in the elderly population. The separation of three factors represented by 16 items results in a short scale, which can be used to monitor the problem of nutritional risk in the elderly environment. However, because this is a preliminary study, carried out in a non-representative group, further testing of this scale is necessary.

## 5. Conclusions

The identification of three nutritional risk factors in a group of older people living in one region in Poland is the result of an adopted and implemented research procedure. These risk factors include unhealthy eating, irregularities related to meals, and perception of body weight. Focusing on irregularities in nutrition and perception of body weight as nutritional risk factors reveals a very narrow perspective. At the same time, the results of previous studies present a comprehensive perspective of nutritional risk. The search for tools to diagnose nutritional risk in older people is becoming necessary due to the difficulty in conducting such studies. The results obtained allowed for developing a 16-item scale. Further testing in a representative group of older people in Poland and other countries is necessary to confirm the possibility of its use in diagnosing nutritional risk among older people.

## Figures and Tables

**Table 1:** Recruitment scheme for respondents.

Place of Study											
Wrocław City						Świdnica District		Wrocław District		Trzebnica District	Sieradz District
Senior organisations participating in the study in each place											
1	2	3	4	5	6	7	8	9	10	11	12
Number of people participating in each place											
36	31	31	30	28	27	34	30	18	14	13	10
Number of people excluded from the study in each place											
0	0	0	0	0	0	0	0	0	0	1	0
Final number of people in the study (N = 301)											

**Table 2:** Socjodemographic and economic characteristics of the study sample.

Variables		N	%
Total		301	100.0
Gender	women	241	80.1
	men	60	19.9
Age (in years)	60–74	219	72.8
	75 and over	82	27.2
Place of residence	village	60	19.9
	city < 100,000 inhabitants	58	19.3
	city > 100,000 inhabitants	183	60.8
Region of residence	City of Wrocław	183	60.8
	Świdnica district	64	21.2
	Wrocław district	32	10.6
	Trzebnica district	12	4.0
	Sieradz district	10	3.4
Education	primary	27	9.0
	basic vocational	49	16.3
	secondary	152	50.5
	higher	73	24.2
Financial situation	below average	26	8.6
	average	241	80.1
	above average	34	11.3
Financial assistance from family	There is no need	245	81.4
	No, despite financial problems	22	7.3
	Yes, due to financial problems	13	4.3
	Yes, despite no financial problems	21	7.0
Social financial assistance	There is no need	259	86.0
	No, despite financial problems	33	11.0
	Yes, due to financial problems	3	1.0
	Yes, despite no financial problems	6	2.0

**Table 3:** Three-factor structure of the EFA-based model.

Factors (F)	Item Number *	Content of Test Items
Unhealthy eating (F1)	13	I usually limit or omit healthy foods from my meals
	14	Usually my meals have little variety (I usually eat the same thing every day)
	15	Usually my meals are not healthy (e.g., I eat fast food).
	24	I eat raw vegetables and fruits less than twice a day
	25	Any vegetables or fruits I eat less than twice during the day (e.g., raw, cooked, pickled)
	49	Prepared store-bought foods (refrigerated or frozen, e.g., soups, dumplings, croquettes, potato noodles, dumplings, etc., which can be quickly cooked or reheated in the microwave) I eat every day
	50	I eat powdered soups or canned or jarred foods, etc., every day
	76	I have trouble choosing healthy foods when shopping
Irregularities related to meals (F2)	7	I eat three or fewer meals a day
	8	I eat six or more meals a day
	9	Breaks between my meals are less than two hours
	10	Breaks between my meals are longer than four hours
Perception of body weight (F3)	3	I would like to increase my body weight
	4	I would like to reduce my body weight
	5	I think my body weight is too high
	6	I think my body weight is too low

* Test item numbers in the survey questionnaire for identifying nutritional risk factors of community-dwelling older people were corrected after the pre-study.

**Table 4:** Measures of fit for the test model.

Measures of Fit *	Factor Model
χ^2^/*df*	3.34
CFI	0.93
TLI	0.92
IFI	0.93
RMSEA	0.06
SRMR	0.09
GFI	0.95
AGFI	0.94

χ^2^/*df*—chi-square fit statistics/degree of freedom, CFI—comparative fit index, TLI—Tucker–Lewis index, IFI—Bollen’s incremental fit index, RMSEA—root mean square error of approximation, SRMR—standardized root mean square residual, GFI—goodness-of-fit statistic, AGFI—adjusted goodness-of-fit statistic, * *p* < 0.05.

**Table 5:** The influence direction of the selected factors on the test items and the results of the estimated coefficients of explained variance (*R*^2^) for the test model.

Factors (F)	Influence Direction	Item Number	Factor Model
Unhealthy eating (F1)	→	13	0.36 *
		14	0.32
		15	0.23
		24	0.43
		25	0.31
		49	0.24
		50	0.25
		76	0.24
Irregularities related to meals (F2)	→	7	0.55
		8	0.52
		9	0.26
		10	0.40
Perception of body weight (F3)	→	3	0.28
		4	0.37
		5	0.69
		6	0.73

→ influence direction of the emerged factor (latent variable) on the test items. * coefficient of determination (*R*^2^).

**Table 6:** Results of estimated reliability and convergent accuracy coefficients for the tested model.

Factors (F)	Factor Model		
	α	CR	AVE
Unhealthy eating (F1)	0.77	0.77	0.30
Irregularities related to meals (F2)	0.75	0.75	0.43
Perception of body weight (F3)	0.79	0.80	0.52

α—Cronbach’s alpha coefficient, CR—composite reliability, AVE—average variance extracted.

**Table 7:** Results of estimated coefficients of differential accuracy (HTMT) for the tested model.

Factors (F)	Factor Model		
	Unhealthy Eating (F1)	Irregularities Related to Meals (F2)	Perception of Body Weight (F3)
Unhealthy eating (F1)	1.00 *	0.16	0.14
Irregularities related to meals (F2)	0.16	1.00	0.06
Perception of body weight (F3)	0.14	0.06	1.00

* HTMT index (heterotrait–monotrait ratio).

**Table 8:** Nutritional risk factors by selected sociodemographic characteristics [% (N)].

Variables	Response Categories	Total	Nutritional Risk Factors					
			F1		F2		F3	
			Intensity					
			Low (N = 149)	High (N = 152)	Low (N = 149)	High (N = 152)	Low (N = 178)	High (N = 123)
Gender	woman	80.1 (241)	88.6 (132)	71.7 (109)	82.6 (123)	77.6 (118)	69.7 (124)	95.1 (117)
	men	19.9 (60)	11.4 (17)	24.3 (43)	17.4 (26)	22.4 (34)	30.3 (54)	4.9 (6)
	*p*-value (chi^2^ test)		*p* = 0.015		*p* = 0.736		*p* < 0.001	
Education	primary	9.0 (27)	6.1 (9)	11.8 (18)	8.1 (12)	9.9 (15)	12.5 (22)	4.6 (5)
	basic vocational	16.3 (49)	14.1 (21)	18.4 (28)	15.3 (23)	17.1 (26)	24.7 (44)	4.1 (5)
	secondary	50.4 (152)	48.3 (72)	52.6 (80)	49.2 (75)	50.6 (77)	49.4 (88)	52.0 (64)
	higher	24.3 (73)	31.5 (47)	17.1 (26)	25.4 (38)	23.4 (35)	13.4 (24)	39.3 (49)
	*p*-value (chi^2^ test)		*p* < 0.001		*p* = 0.844		*p* < 0.001	
Social activity ^1^	often	33.6 (101)	30.9 (46)	36.2 (55)	16.1(24)	50.7 (77)	27.5(49)	42.3 (52)
	sometimes	38.2 (115)	38.3 (57)	38.1 (58)	35.6 (53)	40.8 (62)	34.3 (61)	43.9 (54)
	never	28.2 (85)	26.8 (40)	29.7 (45)	48.3 (72)	8.5 (13)	38.2 (68)	13.8 (17)
	*p*-value (chi^2^ test)		*p* = 0.549		*p* < 0.001		*p* = 0.049	
Family relations ^2^	very good	46.2 (139)	52.3 (78)	40.1 (61)	49.0 (73)	43.4 (66)	50.6 (90)	39.8 (49)
	good	39.2 (118)	42.9 (64)	35.5 (54)	43.6 (65)	34.7 (53)	38.2 (68)	40.6 (50)
	average or worse	14.6 (44)	4.7 (7)	24.3 (37)	7.4 (11)	21.7 (33)	11.2 (20)	19.5 (24)
	*p*-value (chi^2^ test)		*p* = 0.039		*p* = 0.043		*p* = 0.009	

F1—unhealthy eating, F2—irregularities related to meals, F3—perception of body weight, ^1^ Test question: “Do you actively participate in organized (group) meetings, e.g., in various clubs, organizations, associations of a cultural and entertainment nature?”; ^2^ Test question: “How do you assess your relationship with your immediate family, including those with whom you live in the same household?”.

## Data Availability

The data is available in the Knowledge Base of the Wrocław University of Environmental and Life Sciences under the number DOI:10.57755/cbg5-nt66.

## Appendix A

**Table A1:** A description of the nutritional risk situations included in the study questionnaire (97 test items).

Test Item Number	Description of the Situation
1	My body weight has increased in the last 6 months.
2	My body weight has decreased in the last 6 months.
3	I would like to increase my body weight.
4	I would like to decrease my body weight.
5	I think my body weight is too high.
6	I think my body weight is too low.
7	I usually eat three or fewer meals a day.
8	I usually eat six or more meals a day.
9	Breaks between my meals are less than two hours.
10	Breaks between my meals are longer than four hour.
11	I eat irregularly, that is, at different times of the day.
12	I happen not to eat main meals such as breakfast or dinner.
13	I usually limit or omit healthy foods from my meals.
14	Usually, my meals have little variety (I usually eat the same thing every day).
15	Usually, my meals are not healthy (e.g., I eat fast food).
16	Usually, the portions of my meals are too small.
17	Usually, the portions of my meals are too large.
18	I usually eat even if I’m not hungry.
19	I often feel hungry.
20	I rarely feel hungry.
21	I often have an appetite (desire to eat).
22	I rarely have an appetite (reluctance to eat).
23	I usually overeat unhealthy food between meals, such as candy, salty snacks, fast food, etc.
24	I eat fresh (raw) vegetables or fruits less than twice a day.
25	Any vegetables and fruits I eat less than twice a day (e.g., raw, cooked, pickled).
26	I usually eat vegetables and fruits between meals instead of during meals.
27	I eat potatoes or potato dishes (e.g., boiled potatoes, baked potatoes, potato noodles, potato pancakes, etc.) more than once a day.
28	I eat potatoes or potato dishes (e.g., boiled potatoes, baked potatoes, potato noodles, potato pancakes, etc.) rarely or not at all.
29	Grain products (e.g., bread, groats, rice, pasta, etc.) I usually eat more than four times a day.
30	Grain products (e.g., bread, groats, rice, pasta, etc.) I usually eat less than twice a day.
31	I eat whole grain cereal products (e.g., wholemeal or graham bread, dark pasta, coarse groats, brown rice, etc.) once a week or less frequently.
32	I eat processed grain products (e.g., light bread, white rice, fine groats, light pasta, sweet rolls, croissants, etc.) at least once a day.
33	My daily meals include fried foods such as meat or flour dishes.
34	I usually use butter for food preparation or to spread on bread.
35	I usually use margarine or a mix of butter and margarine to prepare meals or spread on bread.
36	I usually use lard or other animal fat (other than butter) to prepare meals or to spread on bread.
37	I usually use oil or olive oil to prepare meals.
38	I eat red meat, fatty cold cuts, offal meats, and processed meat products such as canned goods more than once daily.
39	I eat fish less than twice a week.
40	I eat two eggs less than once a week.
41	I eat two eggs more than once a week.
42	I eat legumes (e.g., peas, broad beans, soybeans, lentils, chickpeas, etc.) less than once a week.
43	I drink two glasses of milk or dairy drinks less than once a day.
44	I usually drink sweetened dairy drinks (e.g., flavoured milk, cocoa, coffee with milk and sugar or honey).
45	I drink natural fermented dairy beverages (e.g., natural yogurt, kefir, buttermilk, etc.) less than once a day.
46	I usually drink sweetened fermented milk drinks (e.g., flavoured yogurt, kefir, buttermilk, etc.).
47	I eat yellow cheeses, including processed and blue cheese, more than once daily.
48	I eat cottage cheese, including homogenised and granular cheese, less than once a day.
49	Prepared store-bought foods (refrigerated or frozen, e.g., soups, dumplings, croquettes, potato noodles, dumplings, etc., which can be quickly cooked or reheated in the microwave) I eat every day.
50	I eat powdered soups or canned or jarred foods, etc., every day.
51	When preparing dishes or eating them at the table, I usually add salt to them.
52	I usually use sugar for cooking and sweetening drinks.
53	I usually drink less than six glasses/cups of water per day, such as filtered tap, spring or mineral water (do not include flavoured waters).
54	I usually drink sweetened hot beverages (e.g., tea, herbal infusions, coffee, etc.)
55	I usually drink more than two cups or two mugs of coffee in a day.
56	I drink sweetened carbonated or non-carbonated drinks every day or almost every day.
57	I drink one glass or one cup of freshly squeezed juice less than once a day.
58	I drink one glass or one cup of sweetened juice “from the carton” or from a bottle more than once a day.
59	I drink energy drinks (such as 2KC, Black Horse, Red Bull, Burn, Shot, and others) more than once a week.
60	I drink strong alcoholic beverages (e.g., pure spirits, whiskey, brandy, etc.) more than once a week.
61	I eat nuts, almonds, and seeds less than once a week.
62	I take vitamin D in tablets or another form, such as liquid, less than once a day.
63	I take various dietary supplements (e.g., vitamins, minerals, omega-3 fatty acids, etc.) without consulting a doctor or nutritionist or reading the leaflet.
64	Instead of a meal, I consume dietary supplements (such as tablets, powder, or liquid).
65	Instead of a meal, I consume special products for nutrition (e.g., shakes, nutri-drinks, bars, etc.).
66	I usually have trouble biting or chewing when consuming a meal or drink.
67	I usually get choked up when consuming a meal or drink.
68	Usually, the consumption of a meal or drink triggers a coughing fit.
69	I usually experience gastrointestinal discomfort while eating a meal.
70	I usually get heartburn during or after a meal.
71	I usually get severe bloating or gas during or after a meal.
72	I usually experience constipation during the day.
73	I usually have diarrhoea after a meal.
74	I usually experience painful bowel movements during the day.
75	I have problems buying food.
76	I have trouble choosing healthy foods when shopping.
77	I have a problem accessing the foods I like.
78	I have difficulty accessing any food.
79	I’m having trouble getting help with food purchases.
80	I have a problem preparing meals at home.
81	I’m having trouble getting help preparing meals at home.
82	I have trouble eating at the table on my own.
83	I eat too little because I don’t like to eat alone.
84	I eat too much because I eat alone.
85	I eat too little because I have health problems that make it difficult for me to leave the house or get to the store for groceries.
86	I eat too little because I have health problems that make it difficult for me to prepare meals.
87	I eat too little because people close to me restrict my access to food.
88	I eat too much because people close to me force me to eat.
89	I eat too little because people close to me do not want to prepare meals for me, even though I have trouble preparing them.
90	I eat too little because I don’t know how to prepare meals.
91	I eat too little because I don’t like to prepare meals.
92	I eat too little because I have trouble eating on my own and in this situation I do not receive help from loved ones.
93	I eat too little because I have trouble eating on my own, and in this situation I do not receive help from the State (e.g., welfare centres).
94	I eat too much because I like to prepare meals.
95	Consumption of drugs reduces my appetite.
96	Consumption of drugs increases my appetite.
97	Consumption of drugs discourages me from eating.
